# Longitudinal characterization of mature beef bull seminal fluid and blood serum metabolome during a 65-day breeding season

**DOI:** 10.1093/tas/txag005

**Published:** 2026-03-12

**Authors:** J D Williams, M S Crouse, M R La Frano, L F Campos-Chillòn, H C Cunningham-Hollinger, C E Field, Z D McFarlane

**Affiliations:** Animal Science Department, California Polytechnic State University, San Luis Obispo, CA, 93410, United States; Meat Animal Research Center, United States Department of Agriculture, Clay Center, NE, 68933, United States; Salk Institute for Biological Studies, Mass Spectrometry Core, La Jolla, CA, 92037, United States; Animal Science Department, California Polytechnic State University, San Luis Obispo, CA, 93410, United States; Department of Animal Science, University of Wyoming, Laramie, WY, 82071, United States; Animal Science Department, California Polytechnic State University, San Luis Obispo, CA, 93410, United States; Animal Science Department, California Polytechnic State University, San Luis Obispo, CA, 93410, United States

**Keywords:** beef bulls, metabolomics, seminal fluid

## Abstract

The metabolic impact of the breeding season on beef bulls has not been extensively studied. The change in plane of nutrition and intensive activity demands induce body weight loss and metabolic changes that may influence fertility and long-term management. The use of metabolomics to characterize the metabolic changes or adaptations during the breeding season will aid in developing hypotheses for future research in beef bulls. The objective of this study was to identify metabolites in both serum and seminal fluid (circulating vs. excretory) that were affected temporally throughout a 65-d breeding season and by bull workload (bull: cow). Over a two-year period, purebred Angus bulls (year 1: *n* = 8, year 2: *n* = 9) owned and housed by the Cal Poly beef unit were surveyed using serum (collected via the coccygeal vein) and seminal fluid (collected via electroejaculation) at the beginning (d0), middle (d30), and end (d65) of a 65-d, post-AI breeding season. Bulls were subjected to one of three workload groups: high (HIGH), moderate (MOD), or low (LOW). Samples underwent HPLC-MS to identify metabolites within the fluids. Data were stratified for sample day and workload and compared using ANOVA for sample day and workload in a 3 × 2 factorial design. Within seminal fluid and blood serum, purine and pyrimidine metabolism pathways were the most affected (*P* *≤* 0.02). Three purine metabolism metabolites showed significant (*P ≤* 0.03) workload × sample day interactions within seminal fluid. Xanthine, hypoxanthine, and inosine showed an increase (*P ≤* 0.03) in abundance in moderate workload bulls throughout the 65-d breeding season and a decrease in abundance in high workload bulls. However, we speculate that a higher workload, likely with more total ejaculates and higher sperm cell turnover, may lead to an increased purine concentration for DNA synthesis. These foundational metabolomic data indicated that an important supplementation strategy to consider may be folate because of the relationship with purine metabolism. Overall, bull workload, particularly a high workload inducing catabolism, may provide a benefit for metabolic health and reproductive performance during the breeding season.

## Teaser

Bull workload during the breeding season primarily impacts purine metabolism in seminal fluid.

## Introduction

Annually, the United States beef industry loses approximately $1 billion to the cost of reproductive failure (Daly and ­Grussing 2021) with estimates ranging from $400-$500 million for both the beef and dairy sector even in the early 2000s, resulting in substantial economic impacts on an annual basis for cattle producers (Bellows et al. 2002). However, this reproductive failure is often attributed solely to females. The interaction of nutrition and reproductive physiology is well studied in female beef cattle, but there is still extensive work necessary in bulls. To combat potential bull infertility in the beef industry, it is recommended that at a minimum, breeding soundness exams (BSE) should be conducted on bull herds annually. Still, more than 20% of California beef producers are not conducting BSE for their breeding bulls (Banwarth et al. 2022) which is similar to the national average of 19.5% (USDA 2020).

Recently, beef bull semen samples have been analyzed at the molecular level, wherein nutritional status affected DNA methylation and could potentially impact semen quality beyond reparation (Moura et al. 2022). The influence of sperm DNA methylation has been linked to sperm cell alteration and infertility in males (Rotondo et al. 2021) and bulls (Costes et al. 2022). These effects in sperm methylation may impact semen quality permanently and could also affect offspring performance. Additional studies have demonstrated clear negative semen quality consequences as an effect of over-nutrition and an anabolic state resulting in reductions in sperm motility and increased morphological defects (Coulter and Kozub 1984; Coulter et al. 1997). However, these consequences were induced by feeding experimental diets to over-condition bulls, thus resulting in negative semen quality outcomes. Recent research analyzing thousands of performance records of university bull tests has indicated that subcutaneous fat accumulation during contemporary group testing resulted in an increase in sperm morphological abnormalities during the first BSE (Smith et al. 2025). Collectively, a growing body of literature has demonstrated the negative consequences of anabolic diets prior to the breeding season that result in reduced semen quality and even poor outcomes in embryonic development (Fontes et al. 2025). However, there are a lack of data collected from breeding beef bulls as they experience a catabolic state during the breeding season. Thus, there is a need for chute side techniques that could identify true biomarkers of infertility in beef bulls resulting in improved BSE performance.

Metabolomics analysis in biological matrices, such as serum and seminal fluid, enables characterization of the metabolic profile associated with different biological states and conditions (Wishart 2019). The presence of metabolites like fructose have been associated with high fertility in dairy bulls (Velho et al. 2018); however, the metabolomic profile of beef bull seminal fluid is relatively unexplored. Metabolomics is a robust diagnostic tool for assessing male infertility and potential biomarkers have been identified in males (Zhou et al. 2016). These metabolic biomarkers may assist in the development of diagnostic tools that could help in promoting accessibility of a more robust BSE for beef producers and large animal veterinarians. Metabolomic assessment may also help elucidate how semen quality and metabolism are affected at different timepoints throughout the duration of the breeding season.

Compilation of a comprehensive profile of metabolites associated with reproductive success will lead to more focused research that can influence and enhance management practices of beef bulls. Furthermore, this profile may provide avenues for strategic supplementation prior to turnout that could help bulls better adapt to the metabolic stress of the breeding season and potentially even impact breeding success. In this study, our objective was to observe metabolic changes within serum and seminal fluid throughout a 65-d breeding season to establish baseline metabolomic profile data for breeding bulls on rangeland. We hypothesized that bulls that experienced greater expected workloads would consequently experience a negative energy balance, leading to reduced body composition and facilitating discovery of potential metabolites of interest for further exploration.

## Materials and methods

### Bull and treatment description

All procedures and protocols were approved by the California Polytechnic State University Institution for Animal Care and Use Committee (protocol number: 19190).

This study utilized purebred Angus beef bulls owned and housed by the California Polytechnic State University, San Luis Obispo Beef Unit over a 2-yr period (Year 1: *n* = 9, Year 2: *n* = 8). Prior to the breeding season, bulls underwent a breeding soundness exam (BSE) conducted by a licensed veterinarian to evaluate readiness for a 65-d, post-artificial insemination breeding season. Breeding soundness exams included a physical examination of overall health (e.g., feet and structural soundness, clearness of eyes, etc), palpation of the testes and accessory sex glands, measurement of scrotal circumference, as well as semen collection and analysis of sperm cell motility, morphology, and concentration. Bulls were tested based on the standards set by the Society of Theriogenology which included the following: non-compromised soundness of structure and limbs, no lameness, met the minimum threshold of scrotal circumference for their age group, minimum 30% progressive motility of sperm, and minimum 70% morphologically normal sperm. (Koziol and Armstrong 2018, Society of Theriogenology, New BSE Manual, Second Edition). Breeding soundness exams were performed before bulls were allocated to one of three workload treatments outlined below. All bulls were classified as satisfactory potential breeders prior to turnout with females.

Bulls were turned out approximately 14-d after timed artificial insemination. Prior to the start of the breeding season, cows and heifers were synchronized using a controlled internal drug-releasing (CIDR) device (Eazi-Breed CIDR, Zoetis Inc., Kalamazoo, MI) with a 7-d CO-Synch + CIDR protocol. On day -7, the females received a single 2 mL intramuscular injection of GnRH (Cystorelin, Merial) and a CIDR. The CIDR was removed on day 0 and a 5 mL intramuscular injection of Prostaglandin (Lutalyse, Zoetis Inc., Kalamazoo, MI) was administered. Approximately 66 h after CIDR removal, females were given a 2 mL intramuscular injection of GnRH (Cystorelin, Merial) and artificially inseminated.

Given the differences among ranch topography and bull: cow ratios on an annual basis, we retroactively stratified bulls based on the following workload classifications based on calf DNA testing records and ranch conditions: (i) high workload (**HIGH**; [bull: cow = 1:42], average pasture size = 28 hectares, average elevation change = 91.44399.29       m), moderate workload (**MOD**; [bull: cow = 1:21], average pasture size = 10.5 hectares, average elevation change = 11 to 40.23 m), and low workload (**LOW**; [bull: cow = 1:10], average pasture size = 20 hectare, average elevation change = 79.25 to 452.93 m). Sampling periods occurred during the start of breeding (d 0), mid-point sample (d 30), and end of the breeding season (d 65).

### Sample collection and processing

Body weights were collected at the beginning and end of the breeding seasons. Body composition characteristics were measured on d 0 and 65 using carcass ultrasound by a licensed Ultrasound Guidelines Council technician (Ultrasound Guidelines Council, Pleasantville, IA; John Openshaw, Modesto, CA). Ultrasound was used to measure intramuscular fat (IMF), marbling (MARB), ribeye area (REA), and subcutaneous rib fat (RIBFAT). Bull activity was measured by average distance traveled per day in kilometers using Knight GPS collars, using the igotU-120 GPS units with a rechargeable battery. Collars with Global Positioning System (GPS) units were placed onto the necks of bulls at the beginning of the breeding season and recorded location at 10-min intervals (Knight et al. 2018). ­Parentage of each calf was tested by a tissue sample that was sent to Neogen Corporation and determined using the SeekSire Parentage program (Neogen Corporation, Lincoln, NE).

Semen was collected via electroejaculation. Ejaculate was aliquoted into 1.5 mL microcentrifuge tubes and snap frozen in liquid nitrogen until further processing (Velho et al. 2018). Once thawed, seminal fluid was centrifuged at 700 RCF for 10 min at 4°C to removed potential debris and sperm cells. To clear remaining sperm cells, supernatant was transferred to another 1.5 mL microcentrifuge tube and centrifuged at 10,000 RCF at 4°C for 60 min. Once completed, supernatant was transferred from the centrifuge tube and stored at −80°C until further processing.

Blood samples were collected via coccygeal venipuncture into serum separating tubes (Corvac, Sherwood Medical, St Louis, MO). Blood was allowed to coagulate at ambient temperature for 10 min before being placed on ice. Blood samples were centrifuged within their collection tubes at 2000 × g for 20 min at 4°C. After centrifugation, serum was separated and transferred to a separate tube and stored at −80°C until further processing.

Prepared samples (serum and seminal fluid) were delivered to the Cal Poly Metabolomics Service Center and underwent ultra-high pressure liquid chromatography—mass spectrometry (UPLC-MS) to analyze metabolite relative abundances as previously described (McMichael et al. 2021). Samples were randomized, de-identified, and assigned new IDs prior to processing and analysis. A semi-targeted metabolomics method screened for polar metabolites confirmed using chemical standards in order to establish retention time and MRM, as well as optimize instrument parameters (Billet 2020). All UPLC-MS analyses, including primary metabolomics, aminomics, and lipidomics, were conducted on a Waters Acquity I-Class UPLC (Waters, Milford, MA, USA) coupled with an API 4000 QTrap (Sciex, Framingham, MA) and quantified with AB Sciex ­MultiQuant version 3.0 in order to generate peak area values. Metabolite abundance was quantified by peak area. All data were normalized to the internal standard 12-([{cyclohexylamino}carbonyl]amino)-dodecanoic acid (CUDA). Quality assurance f analyte peaks was conducted first by manual assessment of peak integrity (such as inspection of peak shape). Following manual assessment, quality assurance was further conducted by measurement of background noise from method blanks, by replicate sample analysis, and by measuring signal intensity from internal standards Additional deuterium and stable isotope labeled internal standards were used to gauge metabolite recovery from samples.

### Forage collection and nutritive value analysis

Forage samples were collected at the respective ranch location of each workload treatment group at days 0, 30, and 65 of each breeding season. Samples were collected via randomized grab sampling using a 0.3 m × 0.3 m quadrant. After collection, samples were labeled with location and date and dried at 60°C for 48 h using a VWR Gravity Convection Oven (VWR International, Radnor, PA, USA). To assess nutritive value of forage available to the bulls during the breeding season, samples were shipped to ServiTech Inc. (ServiTech Inc., Hastings, NE, USA) for quality analysis. Samples were analyzed in duplicate. Forage was analyzed for crude protein (CP) and neutral detergent fiber (NDF). Crude protein content was determined via combustion analysis for total N (Method 976.06 (Horwitz 2000)). Neutral detergent fiber content was determined via ANKOM 200 fiber analysis system (ANKOM Technology Corp., Fairport, NY).

### Statistical analysis

For all analyses, statistical significance was determined when *P ≤* 0.05 and tendencies were determined when *P <* 0.10 but *P >* 0.05. Carcass measurements were stratified by workload and year and tested as a percentage of the initial unit measurement. Carcass measurements were stratified by workload group and tested using ANOVA. Forage CP and NDF were compared between workload treatment groups using ANOVA, between years using t-tests, and a year × workload interaction was analyzed using a generalized linear model (GLM) in JMP Pro, version 16.0. However, this interaction was not significant and was therefore omitted from the model.

Correlations between all carcass characteristics and average distance traveled per day were tested using a Pearson correlation coefficient as well as the number of calves sired by each bull. The correlation between average distance traveled per day and the number of calves sired by each bull was also tested using a Pearson correlation coefficient.

Covariate adjustment was utilized to adjust for any variance between UPLC-MS runs and sample replicates for metabolite abundances and executed in R-studio. Comparison of log-transformed metabolite abundances was performed using ANOVA for data stratified by sample day, and workload group. Differences between the two years were tested using t-tests. Interactions were assessed using a GLM. The three-way interaction of Sample Day × Workload × Year was assessed; however, no significant interaction was expressed and therefore will not be discussed. Analysis of metabolic pathways were assessed in Metaboanalyst, version 5.0 (Pang et al. 2021). Metabolite fluctuation by RIBFAT correlation was conducted using Pearson correlation coefficient. All other statistical analyses were conducted using JMP Pro, version 16.0.

## Results and discussion

### Bull body composition changes, breeding season activity, and calf parentage

Bulls lost an average of 242 kg (25% ± 3.12% of initial weight), 119 kg (10.67% ± 10.2% of initial weight), and 37 kg (3.85% ± 3.74% of initial weight) for HIGH, MOD, and LOW workloads, respectively (Table 1); however, there were no differences in weight loss between treatments (*P = *0.15). Body weight loss may be partially the result of forage nutritive value (Table 2) available to the bulls during the breeding season. There were no significant correlations between the average distance traveled by each bull and their carcass characteristics measured by ultrasound when tested within and across workload groups (*P ≥* 0.35). There was no relationship (*P ≥* 0.10) observed between average distance traveled and the number of calves sired by bull when run within and across workload groups (data not shown; Banwarth 2022). Therefore, bulls who traveled the most, possibly breeding the most cows, did not necessarily sire the most calves. This highlights the importance of a multi-faceted approach to evaluating bull fertility. Methodologies to quantify mounting behavior and an analysis comparing the number of calves sired could aid in better understanding what factors (e.g., behavioral, physiological, etc) make a bull most effective in a multi-sire pasture. Improved methodologies along with current methods of bull evaluation including semen analysis equipment, measures of libido and fitness, and sperm competition with those of other bulls who have bred the same female encompass bull fertility and would aid in improved selection of replacement males.

**Table 1.** Description of workload treatment groups high (HIGH), moderate (MOD), or low (LOW) pastures and the average change in each carcass characteristics as a percentage of the initial measurement.

	Treatment						
	HIGH		MOD		LOW		
Measurement	Mean	SEM	Mean	SEM	Mean	SEM	*P*-value
**Avg. Pasture Size, hectares**	28.0	…	10.5	…	20.0	…	…
**Avg. Elevation change, m**	307.85	…	29.23	…	373.68	…	…
**Bull:Cow^1^**	1:42	…	1:21	…	1:10	…	…
**Carcass characteristics**							
**BW Change, % of initial2**	−25.0	3.12	−10.67	10.2	−3.85	3.74	0.15
** IMF Change, % of initial^3^**	5.73	19.4	−14.98	5.85	11.27	14.4	0.43
** MARB Change, % of initial^4^**	−0.12	3.99	−4.32	1.84	2.61	3.22	0.35
** REA Change, % of initial^5^**	−13.52	3.75	−13.57	3.78	1.94	3.08	0.10
** RIBFAT Change, % of initial^6^**	−60.56^a^	4.36	−43.12^b^	3.69	6.62^c^	19.2	<0.01

1 Ratio of bulls to cows in each treatment group based on DNA confirmation of calving records.

2 Body weight change as a percentage of the initial.

3 Intra-muscular fat as a percentage of the initial.

4 Marbling as a percentage of the initial.

5 Rib-eye area as a percentage of the initial.

6 Rib fat as a percentage of the initial.

a–c Means without a common superscript differ (*P <* 0.05) in main effect of workload.

**Table 2.** Forage nutritive value, measured as crude protein and neutral detergent fiber, compared between workload treatment groups high (HIGH), moderate (MOD), and low (LOW).

	HIGH^a^	MOD	LOW	SEM	*P*-values
**Crude Protein**	10.39	14.99	15.14	1.11	0.0202
**Neutral Detergent Fiber**	60.12	50.97	59.15	1.97	0.1227

a Workload treatments and descriptive data are outlined in Table 1.

There was not a significant relationship between BW change, IMF change, MARB change, and REA change and the number of calves sired by each bull (data not shown, *P ≥* 0.82). However, there was a significant, positive correlation between RIBFAT, and the number of calves sired (data not shown, *P = *0.01, R^2^ = 0.44). In past findings, bulls who were satisfactory versus unsatisfactory based on scrotal circumference and sperm cell motility/morphology, did not differ significantly in the amount of backfat lost (Barth et al. 1995). Bulls in the previously mentioned study were not manipulated for weight loss, similarly to the present study. However, no relationship between backfat loss and breeding soundness was observed (Barth et al. 1995). While we did not observe an effect on our BSE/semen quality metrics, we did observe fluctuation in metabolite abundance that may affect reproductive physiology in either a positive or negative function. We were unable to find any literature illustrating a relationship between subcutaneous fat loss and the number of calves a bull sired. According to our data, subcutaneous fat mobilization was accompanied by an increase in reproductive success. In previous literature, over-conditioning of bulls and excessive adiposity has resulted in diminished semen quality and the physiological stress added due to over-conditioning (Coulter and Kozub 1984; Coulter et al. 1997; Smith et al. 2025; Fontes et al. 2025). Recently, a 5-yr retrospective study of performance and semen quality data from bull development programs in the Southeastern United States indicated a greater percentage of morphologically abnormal sperm cells of bulls in the top 10% and 20% of the contemporary group for subcutaneous fat thickness (Smith et al. 2025). The effect of fat mobilization on bull fertility parameters requires extensive future research. In the meantime, we do not recommend over-conditioning breeding bulls prior to the start of the breeding season. Fat mobilization seems to be important for reproductive success as indicated in the present study; however, metabolic efficiency and utilization efficiency of endogenous metabolic substrates may help bulls more effectively cope with metabolic stress. Selection for bulls that can withstand this metabolic stress during the breeding season may be an important selection factor in the future.

### Metabolic pathway analysis

Following the pathway analysis in Metaboanalyst, there were 3 affected metabolic pathways in both seminal fluid and serum (Table 4; *P ≤* 0.05). In seminal fluid, purine and pyrimidine metabolism were the most affected (*P = *0.01) pathways. The purine metabolism pathway has been studied in beef cattle, reaching beyond the realm of nutrition and reproduction into disciplines as broad as genetics, and stress physiology (Antonelo et al. 2020; Ogunade et al. 2018). According to these data, we can speculate that purine metabolism was affected by the workload assigned to bulls during the breeding season (Table 4). A buildup of the final product of the purine metabolism pathway, urate/uric acid, in excessive amounts results in a disorder known as hyperuricemia. This condition has been related to semen quality wherein hyperuricemia induced a lower total sperm count and semen volume in males (Ma et al. 2022). Pyrimidine metabolism has been described as having the inverse effect as purine metabolism. Studies have observed this pathway leading to an increase in reproductive efficiency (Talluri et al. 2022). The final product of the pyrimidine pathway (uridine) has been described as a potential biomarker of male fertility (Maher et al. 2008). In future research, the uridine mechanism that enhances male fertility should be explored. Researchers have explained that pyrimidine metabolism metabolites, such as uridine, may enhance sperm-cell motility or act as an antioxidant, combatting the negative effects of oxidation (Talluri et al. 2022). Collectively, these data suggest that bulls may require endogenous precursors of the purine metabolism pathway, such as folates, to cope with metabolic stress induced by breeding season activity more effectively. Subclinical deficiency in folic acid and cobalamin reduces gluconeogenesis and fatty acid oxidation efficiency in dairy cows, ultimately reducing metabolic efficiency (Girard and Duplessis 2023). The bulls in the present study received no supplement during the breeding season. However, supplementation of protein, vitamins (such as folic acid which were previously thought to be sufficiently synthesized by rumen microbiota), and minerals may be important to influence energy balance and metabolic efficiency during the breeding season (Crouse et al. 2025).

**Table 3.** The average distance traveled in (km) per day by the bulls was tested using a pearson coefficient correlation in relationship to the average change in carcass characteristics.

	R-Squared	*P*-Value
BW^a^	0.15	0.3454
IMF^b^	0.03	0.6701
MARB^c^	0.07	0.5205
REA^d^	0.03	0.6742
RIBFAT^e^	0.02	0.8091

a Body weight.

b Intra-muscular fat measured via carcass ultrasonography on d0 and d65 of the breeding season.

c Marbling measured via carcass ultrasonography on d0 and d65 of the breeding season.

d Ribeye area measured via carcass ultrasonography on d0 and d65 of the breeding season.

e Subcutaneous rib fat measured via carcass ultrasonography on d0 and d65 of the breeding season.

**Table 4.** Analysis of the metabolic pathways affected by either a high, moderate, or low workload within blood serum and seminal fluid.

Metabolic Pathway	Hits	*P*-value	Impact Factor
**Seminal Fluid**			
** Purine metabolism**	5	0.0020^a^	0.07
** Pyrimidine metabolism**	3	0.0162^a^	0.04
** Arginine biosynthesis**	2	0.0165^a^	0.30
** Glycerophospholipid metabolism**	2	0.0945	0.12
** Arginine and proline metabolism**	2	0.1040	0.07
** Tryptophan metabolism**	2	0.1180	0.13
** Vitamin B6 metabolism**	1	0.1240	0.00
** Phenylalanine metabolism**	1	0.1620	0.00
** Glycerolipid metabolism**	1	0.2100	0.04
** Pantothenate and CoA biosynthesis**	1	0.2440	0.01
** Ether lipid metabolism**	1	0.2550	0.00
** Pyruvate metabolism**	1	0.2770	0.00
** Propanoate metabolism**	1	0.2880	0.00
** Glycolysis/gluconeogenesis**	1	0.3190	0.00
** Glycine, serine and threonine metabolism**	1	0.3960	0.00
** Valine, leucine, and isoleucine degradation**	1	0.4480	0.02
** Primary bile acid biosynthesis**	1	0.4960	0.02
** Aminoacyl-tRNA biosynthesis**	1	0.5110	0.00
**Blood serum**			
** Pyrimidine metabolism**	3	0.0162^a^	0.04
** Primary bile acid biosynthesis**	3	0.0271^a^	0.07
** Glycolysis/gluconeogenesis**	2	0.0532^a^	0.00
** Synthesis and degradation of ketone bodies**	1	0.0708	0.00
** Glycine, serine and threonine metabolism**	2	0.0857	0.00
** Glycerophospholipid metabolism**	2	0.0945	0.00
** Amino sugar and nucleotide sugar metabolism**	2	0.0991	0.07
** Taurine and hypotaurine metabolism**	1	0.1110	0.00
** Tryptophan metabolism**	2	0.1180	0.00
** Phenylalanine metabolism**	1	0.1620	0.23
** Butanoate metabolism**	1	0.1980	0.00
** Pantothenate and CoA biosynthesis**	1	0.2440	0.00
** Purine metabolism**	2	0.2490	0.01
** Selenocompound metabolism**	1	0.2550	0.00
** Ether lipid metabolism**	1	0.2550	0.00
** Pyruvate metabolism**	1	0.2770	0.00
** Propanate metabolism**	1	0.2880	0.00
** Galactose metabolism**	1	0.3290	0.05
** Alanine, asparate and glutamate metabolism**	1	0.3390	0.00
** Arginine and proline metabolism**	1	0.4310	0.01
** Aminoacyl-tRNA biosynthesis**	1	0.5110	0.00

a Indicates statistical significance at *P* < 0.05

### Metabolite analysis

In seminal fluid, 28 metabolites were identified in sufficient quality (Table 6). There was a significant sample day × workload interaction for hypoxanthine (*P = *0.02), an intermediate of purine metabolism. Hypoxanthine was observed in increasing amounts from d0 to d65 in the MOD workload group, decreasing amounts in the HIGH workload group, and unchanged in the LOW workload group. In past research, hypoxanthine has been identified as a biomarker of the onset of hyperuricemia (Kostalova et al. 2015). This disorder presents as asymptomatic in females, but in males has been known to be associated with neurological issues and infertility (Ma et al. 2019). Another metabolite within the purine metabolism pathway is inosine, which also displayed a significant sample day × workload interaction in the dataset (*P = *0.02). Inosine was observed in increasing amounts from d0 to d65 in the MOD workload group, decreasing amounts in the HIGH workload group, and unchanged in the LOW workload group. Inosine has been described in relation to oxidative stress within a human male ejaculate, causing increased reproductive failure (Rosenfeld 2018). Oxidative stress in semen leads to a rapid loss of ATP in the sperm cells, causing decreased motility, and increased sperm cell morphological abnormalities (Ma et al. 2019). These data related to reactive oxygen species suggest the need for a more comprehensive investigation of selenocompound metabolism and the transsulfuration pathway. From a more practical standpoint, a more thorough assessment of changes in concentration of selenium, Vitamin E, and other downstream metabolites may be important to investigate in the future. A survey of mineral concentration in whole blood and serum of California beef cattle herds were found to be below the critical threshold in selenium, copper, zinc, and manganese at levels of 12%, 28%, 36%, and 92%, respectively (Davy et al. 2019).

Xanthine has been correlated to sperm abnormalities and low motility in human males (Sanocka et al., 1997). There was a significant sample day × workload interaction for xanthine within the seminal fluid of the bulls. Xanthine was observed in increasing amounts from d0 to d65 in the MOD workload group (Mean = 4.83; SD = 0.45), decreasing amounts in the HIGH workload group (Mean = 4.42; SD = 0.64), and unchanged in the LOW workload group (Mean = 4.42; SD = 0.64). Hypoxanthine, inosine, and xanthine are all intermediates of the purine metabolism pathway. The purine metabolism pathway was the most significantly affected pathway within seminal fluid (*P = *0.01), and all three of these metabolites displayed significant sample day × workload interactions. Within workload groups, we saw increased relative abundances of each of these metabolites within the LOW workload group compared with the HIGH and MOD workload groups. According to these data, purine metabolism may be down regulated in bulls experiencing higher expected workloads. We speculate that folate may be limiting as a consequence of the elevated nutritional requirements for bulls in the high workload group, thus resulting in the observed disruption of the purine metabolism pathway.

The fluctuation of seminal fluid metabolites showed no correlation to the change in carcass characteristics BW, IMF, MARB, and REA (Table 7; *P ≥* 0.10). However, four seminal fluid metabolites showed an apparent relationship to RIBFAT fluctuation. This includes 4-pyridoxate, cytidine, guanosine, and salicylurate (*P ≤* 0.05). Seven seminal fluid metabolites showed a significant correlation with change in RIBFAT within one of the three workload groups (*P ≤* 0.05). There were correlations within each treatment group, but no metabolite showed a significant relationship within more than one treatment group. This indicates the specificity of each metabolite in its relationship to loss in body composition of beef bulls, supporting our hypothesis that bull experiencing differing workload expectations would experience separate metabolic effects.

**Table 5.** Interactions between workload groups compared with year and day in seminal fluid.

Metabolite by like compounds	*P*-value	*P*-value
	Workload * Year	Workload * Day
**Purine metabolism**		
** Xanthine**	0.8298	0.0231^a^
** Hypoxanthine**	0.7572	0.0270^a^
** Inosine**	0.6882	0.0236^a^
** Guanosine**	0.3724	0.1319
** Urate**	0.1437	0.8190
**Pyrimidine metabolism**		
** Uridine**	0.6370	0.0667
** Cytidine**	0.7559	0.0703
** Deoxycytidine**	0.3145	0.0752
**Arginine biosynthesis**		
** Arginine**	0.9527	0.6362
** Citrulline**	0.8343	0.2359
**Glycerophospholipid metabolism**		
** Alpha-glycerophosphocholine**	0.4469	0.0542^a^
**Arginine and Proline metabolism**		
** Creatine**	0.0341^a^	0.9041
**Tryptophan metabolism**		
** 5-hydroxytryptophan**	0.0803	0.2481
** Anthranilic acid**	0.8570	0.4066
**Other**		
** 4-pyridoxate**	0.0345^a^	0.5134
** Alpha-glycerophosphate**	0.6539	0.0254^a^
** Alpha-hydroxybutyrate**	0.6380	0.0693
** Glycocholate**	0.6928	0.1764
** Glycodeoxycholate**	0.6206	0.2050
** Hippurate**	0.2667	0.0109^a^
** Lactate**	0.8310	0.0556
** Methylmalonate**	0.9860	0.0651
** Pantothenate**	0.4928	0.8984
** Salicylurate**	0.1229	0.8984
** Aminoisobutyric acid**	0.8014	0.2937
** Creatinine**	0.4766	0.7844
** Cyclic AMP-cAMP**	0.2789	0.5566
** Dimethyl-L-arginine ADMA**	0.9766	0.3878

a Indicates statistical significance.

**Table 6.** Metabolites detected within seminal fluid, compared by workload treatment group (either high [HIGH], moderate [MOD], or low [LOW]), and sample day (comparing sample days 0, 30, or 65).

	LOW^1^			MOD			HIGH				*P*-value	
Metabolite by like compounds	d0^2^	d30	d65	d0	d30	d65	d0	d30	d65	SEM	Day	Workload
**Purine metabolism**												
** Xanthine**	4.94	4.54	5.09	3.99	4.31	4.95	4.46	4.73	4.15	0.17	0.3704	0.1510
** Hypoxanthine**	5.86	6.00	6.29	4.75	4.8	5.91	5.5	5.35	4.82	0.21	0.4126	0.0103^a^
** Inosine**	6.06	6.27	6.47	4.85	4.65	6.14	5.59	5.37	4.67	0.25	0.5594	0.0090^a^
** Guanosine**	5.07	5.10	4.97	4.23	4.44	5.01	4.63	4.88	4.45	0.15	0.5814	0.0770
** Urate**	4.18	4.34	4.29	3.74	3.81	4.16	4.02	3.91	4.02	0.15	0.5996	0.1084
**Pyrimidine metabolism**												
** Uridine**	4.83	4.48	4.69	3.88	4.07	4.65	4.22	4.33	4.03	0.14	0.6075	0.0329^a^
** Cytidine**	4.87	4.79	4.98	4.15	4.37	4.83	4.46	4.81	4.23	0.14	0.4838	0.0634
** Deoxycytidine**	5.35	5.64	5.5	4.68	5.17	5.61	5.15	5.5	4.9	0.17	0.6650	0.1579
**Arginine biosynthesis**												
** Arginine**	5.64	5.51	5.54	5.44	5.76	5.94	5.54	6.19	5.74	0.17	0.3612	0.5475
** Citrulline**	4.74	4.47	4.81	4.27	4.63	4.77	4.56	4.85	4.63	0.12	0.3251	0.5747
**Glycerophospholipid metabolism**												
** Alpha-glycerophosphocholine**	7.07	7.65	7.74	6.12	5.83	7.15	7.31	6.91	6.08	0.29	0.9061	0.0316^a^
**Arginine and Proline metabolism**												
** Creatine**	6.18	6.41	6.44	6.17	6.25	6.49	6.14	6.63	6.28	0.14	0.1753	0.9980
**Tryptophan metabolism**												
** 5-hydroxytryptophan**	3.9	4.06	3.97	4.08	3.93	4.03	3.85	4.16	4.33	0.09	0.1783	0.4481
** Anthranilic acid**	4.36	4.28	4.98	4.52	4.8	5.08	4.62	4.87	4.81	0.17	0.0361a	0.3218
**Other**												
** 4-pyridoxate**	6.75	6.40	6.90	6.00	6.10	6.71	6.32	6.13	6.29	0.17	0.1492	0.0648
** Alpha-glycerophosphate**	6.67	6.91	7.23	5.69	5.42	6.63	6.81	6.33	5.61	0.24	0.7251	0.0311^a^
** Alpha-hydroxybutyrate**	4.82	4.58	5.06	4.59	4.75	5.09	4.74	5.19	4.68	0.15	0.3310	0.9089
** Glycocholate**	3.87	3.04	3.69	3.71	3.78	3.94	3.76	4.03	3.92	0.16	0.5043	0.1360
** Glycodeoxycholate**	3.97	3.43	4.01	3.81	4.18	4.24	3.53	4.19	3.98	0.17	0.3060	0.4338
** Hippurate**	5.46	6.53	5.45	6.53	7.17	6.5	5.2	6.61	6.88	0.23	0.0004a	0.0597
** Lactate**	6.5	6.44	6.91	6.17	6.44	6.7	6.37	6.51	6.02	0.13	0.4571	0.1715
** Methylmalonate**	6.38	6.02	6.58	6.04	6.11	6.37	6.28	6.15	6.00	0.11	0.0967	0.3924
** Pantothenate**	4.81	4.83	5.23	4.54	4.78	4.99	4.58	4.89	4.8	0.17	0.1858	0.5037
** Salicylurate**	5.54	5.55	5.29	6.18	5.92	6.09	5.46	5.3	5.6	0.19	0.8999	0.0030^a^
** Aminoisobutyric acid**	5.29	5.27	5.26	5.09	4.92	5.26	5.37	5.09	5.1	0.07	0.6577	0.4106
** Creatinine**	6.46	6.89	6.54	6.86	7.14	6.96	6.59	7.19	7.0	0.14	0.0175	0.0578
** Cyclic AMP-cAMP**	3.57	3.82	3.72	3.93	4.08	3.87	3.52	4.31	4.1	0.16	0.0926	0.3465
** Dimethyl-L-arginine ADMA**	5.7	5.47	5.61	5.70	5.85	6.03	6.28	5.74	5.9	0.14	0.2072	0.2167

a Indicates statistical significance.

1 Workload treatments descriptive data are outlined in Table 1.

2 Samples collected at the beginning (d0), middle (d30), and end (d65) of the breeding season.

**Table 7.** Pearson correlations between change in subcutaneous rib fat as a percentage of the initial measurement and the change in metabolite relative abundance by workload treatment group, either (HIGH), moderate (MOD), or low (LOW).

	HIGH^1^		MOD		LOW	
	R-squared	*P*-value	R-squared	*P*-value	R-squared	*P*-value
**Blood serum metabolites**						
** Glycocholate **	0.72	0.0689	0.13	0.5539	0.23	0.5163
** Guanosine **	0.12	0.118	0.02	0.8233	0.32	0.3212
** Inosine **	0.61	0.1167	0.24	0.3973	0.05	0.7805
** Pantothenate **	0.01	0.8522	0.08	0.6439	0.76	0.1289
** Creatine **	0.05	0.7196	0.11	0.5864	0.01	0.9595
**Seminal Fluid Metabolites**						
** 4-Pyridoxate **	0.03	0.7812	0.88	0.0191^a^	0.01	0.9899
** Alpha-Glycerophosphate **	0.51	0.1760	0.44	0.2249	0.68	0.1779
** Cytidine **	0.16	0.5048	0.78	0.0481^a^	0.59	0.2347
** Guanosine **	0.85	0.0252^a^	0.18	0.4775	0.91	0.0478^a^
** Hippurate **	0.01	0.8561	0.32	0.3164	0.27	0.4777
** Hypoxanthine **	0.39	0.2567	0.21	0.4434	0.86	0.0731
** Inosine **	0.55	0.1525	0.51	0.1742	0.73	0.1468
** Salicylurate **	0.01	0.9858	0.59	0.1273	0.95	0.0229^a^
** Uridine **	0.17	0.4851	0.63	0.1087	0.72	0.1515
** Alpha-Glycerophosphocholine **	0.58	0.1350	0.51	0.1743	0.70	0.1615
** Creatinine **	0.13	0.5531	0.16	0.5016	0.26	0.4873
** Cytidine **	0.27	0.3668	0.82	0.0340^a^	0.93	0.0371^a^

a Indicates statistical significance.

1 Workload treatment groups and descriptive statistics are outlined in Table 1.

**Table 8.** Metabolites detected within blood serum, compared by workload treatment group (either high [HIGH], moderate [MOD], or low [LOW]), and sample day (comparing sample days 0, 30, or 65).

	LOW^1^			MOD			HIGH				*P*-values	
Metabolite by like compounds	d0^2^	d30	d65	d0	d30	d65	d0	d30	d65	SEM	day	workload
**Pyrimidine metabolism**												
** Uridine**	4.57	4.91	4.77	4.71	4.45	4.63	4.55	4.82	4.62	0.10	0.3697	0.1989
** Cytidine**	4.59	4.81	4.71	4.6	4.49	4.63	4.54	4.72	4.68	0.06	0.2378	0.9664
** Deoxycytidine**	5.18	5.4	5.62	5.17	5.37	5.49	5.23	5.23	5.26	0.09	0.0002^a^	0.8387
**Primary bile acid biosynthesis**												
** Taurochenodeoxycholate**	4.14	4.36	4.32	3.84	3.85	4.18	3.74	4.13	4.24	0.14	0.6552	0.4272
** Glycocholate**	5.99	6.46	6.02	5.99	5.68	5.96	6.1	6.08	6.23	0.15	0.7478	0.0436^a^
** Taurocholate**	5.59	5.85	5.85	5.24	5.14	5.66	5.33	5.59	5.43	0.58	0.2045	0.1784
**Glycolysis/gluconeogenesis**												
** Lactate**	6.91	7.15	7.3	7	7.02	6.95	6.95	7.18	6.94	0.09	0.5637	0.8142
** Fructose, Glucose, Galactose**	6.3	6.37	6.13	6.28	5.83	5.87	6.19	6.1	6.16	0.1	0.3519	0.5083
**Glycine, Serine, and Threonine metabolism**												
** Choline**	6.87	6.97	7.13	6.84	6.93	7.11	6.87	6.88	6.83	0.08	0.0048^a^	0.4669
** Creatine**	6.94	6.98	7.26	6.89	6.99	7.13	6.95	6.89	6.91	0.09	0.0013^a^	0.0754
**Tryptophan metabolism**												
** 5-hydroxytryptophan**	5.06	5.33	5.81	5.13	5.51	5.91	4.84	5.19	5.14	0.21	0.0564	0.7614
** Kynurenine**	4.72	4.81	5.31	4.67	5	5.3	4.79	4.67	4.76	0.17	0.0159^a^	0.6552
**Purine metabolism**												
** Inosine**	4.62	4.97	4.49	5.01	4.38	4.64	4.72	4.99	4.91	0.21	0.1329	0.0040^a^
** Guanosine**	4.08	4.52	4.32	4.59	4.26	4.63	4.41	4.69	4.48	0.13	0.3316	0.0015^a^
**Other**												
** Beta-hydroxybutyrate**	5.91	5.8	6.32	6.08	6.05	6.27	5.75	5.93	5.81	0.13	0.0038^a^	0.3161
** Hippurate**	6.66	6.66	6.89	6.77	6.71	7.03	6.68	6.68	6.75	0.09	0.0863	0.5536
** Pantothenate**	4.72	4.78	5.02	4.9	4.56	4.87	4.64	4.74	4.68	0.09	0.5530	0.0655
** Alanine**	5.98	6	6.14	5.9	5.89	6.04	5.9	5.96	5.89	0.06	0.0089^a^	0.5591
** Alpha-glycerophosphocholine**	4.86	4.98	5.4	4.98	4.97	5.24	4.96	5.02	4.94	0.09	0.0342^a^	0.7202
** Dimethyl-L-arginine ADMA**	6.24	6.26	6.48	6.16	6.19	6.36	6.11	6.22	6.12	0.07	0.0113^a^	0.7132

a Indicates statistical significance.

1 Workload treatment groups and descriptive data are outlined in Table 1.

2 Samples collected at the beginning (d0), middle (d30), and end (d65) of the breeding season.

**Fig. 1.**
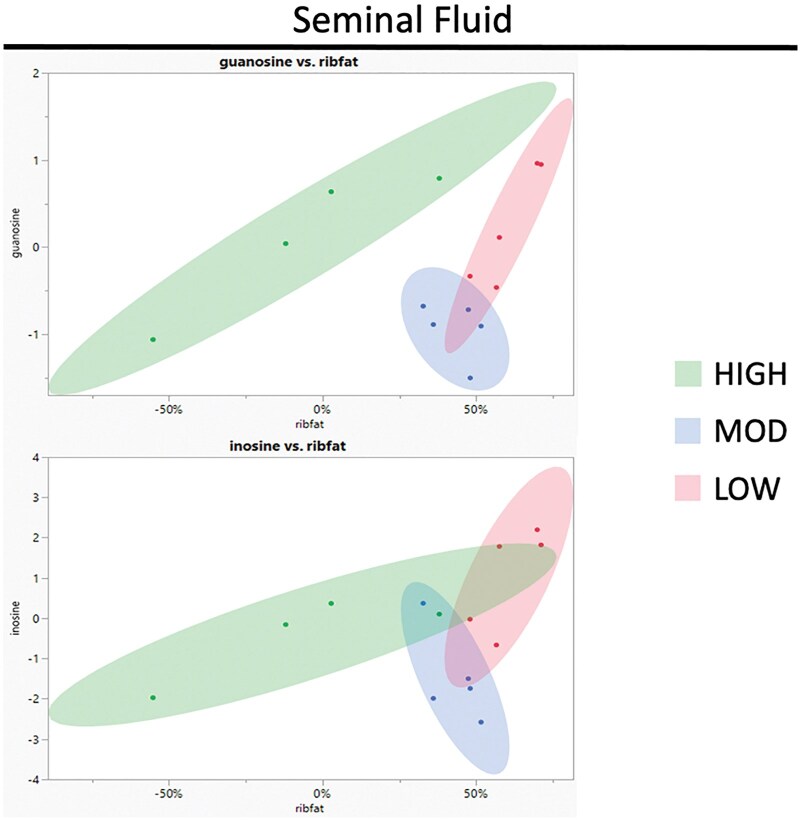
Principle component analysis (PCA) plots illustrating the relationship, tested by pearson coefficient analysis, between bull subcutaneous rib fat loss during the breeding season and change in metabolite relative abundances in seminal fluid. Workload groups (HIGH, MOD, LOW) and descriptive data are outlined in Table 1.

**Fig. 2.**
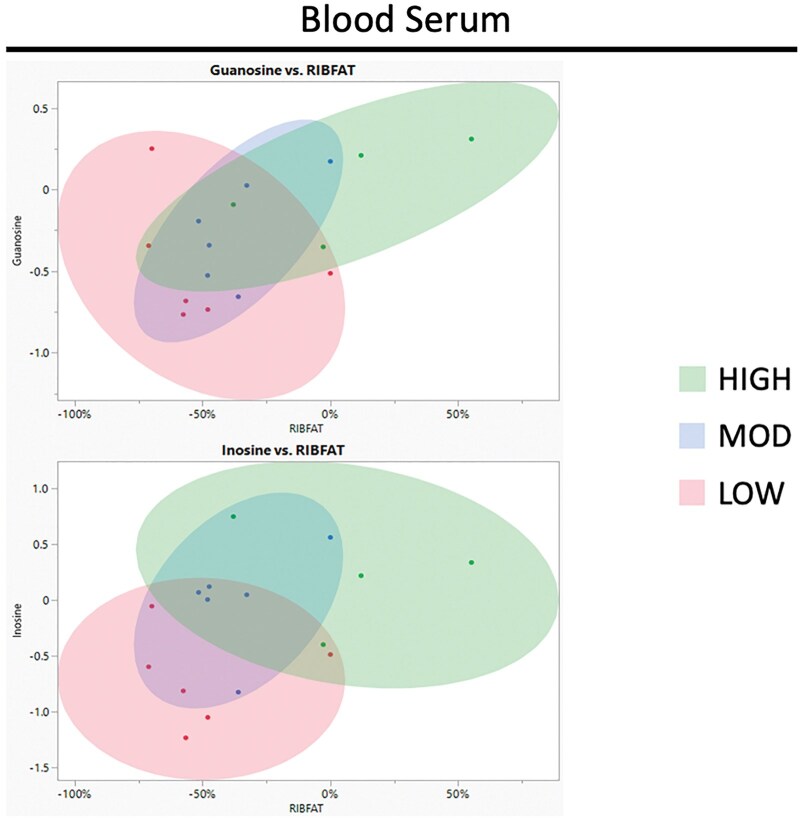
Principle component analysis (PCA) plots illustrating the relationship, tested using a pearson coefficient analysis, between bull subcutaneous rib fat loss during the breeding season and change in metabolite relative abundances in blood serum. Workload groups (HIGH, MOD, LOW) and descriptive data are outlined in Table 1.

Principal component analysis (PCA) of metabolites and their correlation to RIBFAT is illustrated in Fig. 1. When comparing the seminal fluid metabolite PCA plots (Fig. 1) to the blood serum PCA plots (Fig. 2), seminal fluid metabolites displayed clearer separation between the workload treatment groups. Thus, indicating a closer relationship between subcutaneous fat loss and the seminal fluid metabolome when compared with the blood serum metabolome. The effect of subcutaneous fat on the quality of semen has been researched previously. By manipulating nutrition level, scientists investigated the effects of changing body composition on the quality of an ejaculate (Harrison et al. 2022). In this study, scientists did not observe an effect on sperm motility but saw a significant increase in sperm cell morphological abnormalities in the over-conditioned bulls. Therefore, it was not surprising that loss of RIBFAT showed a close relationship to metabolites within seminal fluid.

In serum, 21 metabolites were identified (Table 5). Most blood serum metabolites experienced a year effect, which may be explained by the difference in nutritive value (NDF and CP) of the forage available to the bulls during the breeding season. Within the serum metabolites, the pyrimidine metabolism pathway was the most significantly affected pathway (Table 4). However, when accounting for day and workload effects, metabolites inosine and guanosine, involved in the purine metabolism pathway, indicated a significant day effect (*P* < 0.05; Table 8). The purine metabolism pathway was also implicated in the previously presented seminal fluid results, thus indicating the potentially important role of purine metabolism for bulls coping with the metabolic stress of the breeding season. The pyrimidine pathway was also indicated as another metabolic pathway of interest in the previously discussed Metabolic pathway analyses for seminal fluid and serum. In complementary studies, scientists have claimed that low concentrations of uridine (final product of the pyrimidine pathway) may be a potential biomarker of human male infertility (Maher et al. 2008). Pyrimidine metabolism metabolites cytidine and deoxycytidine both were in higher relative abundance in the HIGH and MOD groups when compared with the LOW group. This was consistent with analysis of the seminal fluid metabolites, where bulls that experienced higher expected workloads potentially benefited from a higher workload treatment physiologically.

In summary, these data indicated physiological changes experienced by beef bulls during the breeding season that correspond to decreased body energy reserves. Purine and pyrimidine metabolism have established relationships with semen quality in males of multiple species. Significant impact on these pathways was demonstrated within the blood serum and semen metabolome of the bulls utilized in this study. The final products of purine and pyrimidine metabolism are urate and uridine, respectively. This pathway has proven effects in the reproductive physiology of beef bulls as previously discussed. The development of a chute side test, that can be conducted in correspondence with a BSE would benefit producers and veterinarians, allowing them to select bulls that would be most effective to cope with the metabolic stress of the breeding season. While uridine has been associated with a positive impact on semen quality, urate has had the opposite effect. Expression of purine metabolism metabolites (xanthine, hypoxanthine, and inosine) demonstrated in the present study decreased relative abundances in high workload bulls as the 65d breeding season progressed. Change in pyrimidine metabolism metabolites did not show significant interaction in a sample day × workload interaction; however, the impact of the pathway when influenced by body weight or subcutaneous fat loss should be studied further. Bull dominance and competition likely also played a role in the current dataset and should be another factor considered in future research. Based on this foundational dataset, folate may be an important supplement to consider providing bulls before, during, and after the breeding season as a result of the relationship with purine metabolism. Collectively, these data indicated that workload influenced metabolic health, and a high workload could be beneficial for overall metabolic and reproductive health.
